# Comparative tests of ectoparasite species richness in seabirds

**DOI:** 10.1186/1471-2148-7-227

**Published:** 2007-11-15

**Authors:** Joseph Hughes, Roderic DM Page

**Affiliations:** 1 Department of Environmental and Evolutionary Biology, University of Glasgow, UK

## Abstract

**Background:**

The diversity of parasites attacking a host varies substantially among different host species. Understanding the factors that explain these patterns of parasite diversity is critical to identifying the ecological principles underlying biodiversity. Seabirds (Charadriiformes, Pelecaniformes and Procellariiformes) and their ectoparasitic lice (Insecta: Phthiraptera) are ideal model groups in which to study correlates of parasite species richness. We evaluated the relative importance of morphological (body size, body weight, wingspan, bill length), life-history (longevity, clutch size), ecological (population size, geographical range) and behavioural (diving versus non-diving) variables as predictors of louse diversity on 413 seabird hosts species. Diversity was measured at the level of louse suborder, genus, and species, and uneven sampling of hosts was controlled for using literature citations as a proxy for sampling effort.

**Results:**

The only variable consistently correlated with louse diversity was host population size and to a lesser extent geographic range. Other variables such as clutch size, longevity, morphological and behavioural variables including body mass showed inconsistent patterns dependent on the method of analysis.

**Conclusion:**

The comparative analysis presented herein is (to our knowledge) the first to test correlates of parasite species richness in seabirds. We believe that the comparative data and phylogeny provide a valuable framework for testing future evolutionary hypotheses relating to the diversity and distribution of parasites on seabirds.

## Background

Avian ectoparasitic lice have a widespread geographic and host distribution, making them excellent models for exploring the ecological and evolutionary dynamics of host-parasite associations. However, little is known about which host factors influence avian louse diversity. Comparative studies on correlates of parasite species richness have mainly focused on mammals [1-3] and have revealed host range size, body mass and diet as factors influencing parasite species richness [4]. Other studies have also found support for an effect of host basal metabolic rate [5], geographic latitude [6] and population density [2]. Species richness of avian parasites is also known to co-vary with host body size, range size, habitat, phylogeny, latitude and immune defences [7,8]. However, work specific to ectoparasites showed that tropical birds have a similar louse diversity to temperate species and did not find a correlation between louse species richness and any of the 13 host variables examined (e.g., host body size, density, geographic range, microhabitat use, standard dimensions of bill, foot and toenail morphology, etc) after controlling for sampling effort and phylogeny [9]. Additionally, patterns of correlation vary between the two lice suborders. Møller and Rózsa [10] showed that host immune response does influence lice diversity in the amblyceran lice, which feed on skin and blood, but not in ischnoceran lice, which live on feathers and feed on keratin.

The studies on avian lice have been mainly focused on land birds and altricial species (passerines, woodpeckers, owls, etc). In this paper we focus on seabirds and a broad range of host morphological characters, life history traits, and extrinsic factors such as geographic range. The seabird-louse system offers some unique opportunities, as there are three distinct clades of "seabirds": the Procellariiformes (albatrosses and petrels), Pelecaniformes (gannets, boobies, cormorants, shags, pelicans and frigate birds), and the Charadriiformes (gulls, skuas, auks and their allies); and these birds are host to both suborders of lice (Ischnocera and Amblycera). The presence of these "ecological replicates" [11] permits stronger tests of hypotheses concerning correlates of parasite diversification, because we have multiple lineages available for testing. In view of the disparate phylogenetic histories of the Ischnocera and Amblycera, and clear ecological differences between these two suborders [10,12], louse diversity was measured with the suborders separated, and combined (total lice species richness). Classical taxonomic practice assumed a high degree of host specificity [13], although some described lice are believed to infect hosts from various avian families on multiple continents. Thus, we used a fourth measure of richness, i.e. genera richness, to partly resolve the problem of overestimating taxonomic richness due to the uncertainty of morpho-species. Indeed, recent molecular analyses of *Dennyus *lice based primarily on mitochondrial sequences have revealed a wealth of genetic diversity among parasite lineages that is not always apparent in their morphology [14,15]. Including parasite phylodiversity would provide a more objective means of measuring parasite richness [16] but this method would entail a substantial reduction in our sample size due to insufficient phylogenetic sampling of the Phthiraptera and thus was not included in this study. We collected data to determine whether our four measures of lice richness (Amblyceran richness, Ischnoceran richness, total lice species richness, lice genera richness) were influenced by the following sets of factors.

### Host morphology and mass

While the plumage of a host bird seems like a uniform environment, it is actually a series of interconnected microhabitats partitioned by the different feather types present on the wings, back, head, and rump. Different species of lice are morphologically and behaviourally adapted to exploiting these niches on their host [17], hence several species of lice may coexist on the same host species. As larger-bodied hosts represent a larger surface area and probably offer more niches for colonization [8,18], the diversity of lice is expected to increase with host body mass. Similarly, longer wings and larger bodies are expected to provide a larger number of niches for lice to colonize. The differential ability of birds to preen or groom various parts of their bodies also exerts a major selection pressure on louse which may affect their diversity on a host. One study has shown a correlation between the fine structure of the bill tip and louse abundance [9]. Since preening by the bill tip plays a major role in avian defense against lice [19], measures of bill morphology may also co-vary with measures of louse richness in birds. Birds with shorter bills are likely to be more able to preen than larger billed species like the pelican and therefore likely to have a lower lice species diversity.

### Life history

Longer-lived hosts could harbour greater parasite diversity because they encounter more parasite species during their lifetimes [20,21] and host species with lower mortality could also increase the ability of parasites to become established on a host population [22]. Small clutch sizes are also predicted to reduce parasite prevalence and limit parasite establishment. Indeed, investigators have argued that species that live in conditions with increased abundance of ectoparasites should evolve reduced clutch size [23,24]. This is likely to be the case for hosts infested with lice (generation time of approximately 21 days for wing lice [25]), whereas ectoparasites with long generation times are likely to favour increased clutch size [24,26,27].

### Total population size and geographic range

Interspecific and intraspecific interactions generate a network through which parasites spread within or between species [28]. Factors that increase the parasite's reproductive success, such as host population density, rates of among-host contact, and encounter rates with parasites, should correlate positively with parasite species richness. Recent studies have confirmed host density as a significant predictor of parasite richness in mammals [29], and similar results may be expected with total host population size [2] as the size of the parasite community may be influenced through island biogeographic effects (larger populations corresponding to larger island habitats). A larger host population would increase the chances of colonization and would provide more resources for exploitation by the colonizing parasite. The geographic range of a host may also influence patterns of parasite species richness if the risk of being colonised by a louse varies among geographical locations, or if a host is exposed to a wider diversity of habitats at the population and species level. A host species with a larger geographical range may occupy more different habitats, or come into contact with a larger number of other species, leading to higher parasite species richness [30-32]. Additionally, a larger geographical range may indicate that a species has a larger number of host individuals, increasing the likelihood that more parasites become established [33].

### Bird behaviour

Host behaviour may also play a role in determining the parasite richness. For example, Felsõ and Rózsa [34] showed that lice genera richness was significantly lower in diving birds in contrast to non-diving birds. They put forward three hypotheses to explain these differences: (1) the louse richness is affected by the presence of water, (2) the plumage of diving and non-diving birds differ and (3) the preen-oil may differ between the two diving behaviours. These findings will be further tested with our dataset. Other behavioural observations such as time spent preening and nesting density may also affect the ectoparasite richness, unfortunately these types of behavioural observations are either rarely reported in the literature or inconsistently measured between species.

### Host diversification

A number of studies have shown that at least some seabirds and their lice do cospeciate [13,35,36]. This coevolutionary interaction between lice and their hosts could have increased the diversity of lice as a result of specialization onto their diversifying hosts. However, despite the presumed importance of the role of coevolutionary interactions in diversification, the evidence is limited [3,11,37]. In this study, we test whether the seabird diversification and lice diversity are correlated and the extent to which louse diversity varies across the three seabird orders.

To summarise the predictions, we expect more parasites on large long-lived and non-diving birds with short bills, large clutch sizes, large geographical ranges and large population sizes. These predictions are likely to co-vary. Additionally, we predict that there will be more parasites on more diverse groups of hosts. By focusing on three clades of seabirds and including multiple predictor variables, we can attempt to distinguish among confounding or correlated factors. Moreover, studying both Ischnocera and Amblycera together and separately may reveal the differences in patterns specific to one group as well as patterns applicable to all lice. However, if cospeciation is prevalent, host phylogeny is likely to be at least as important as host ecology in determining the composition of the parasite community, because the parasite community of a host species has likely been inherited from its ancestor, hence we also incorporated phylogenetic information in our analyses by using independent contrasts.

## Results

### Lice diversity on seabirds

The variables used for the analyses and information on sample size are summarised in Table 1, (see also Addtional Files 1, 2 and 3). Population size was normally distributed according to the Kolmogorov-Smirnov one sample test (D = 0.0611, p-value = n.s.) and geographic range was left-skewed (D = 0.2662, p-value < 0.01). A total of 440 different lice species from 37 genera were found on the 413 bird species. The distribution of parasites was highly aggregated, with most birds having 2 or fewer parasites and a few hosts having 10 or more parasitic lice. As expected we found a strong correlation between the sampling effort to which each bird species was studied and the number of parasitic lice species recorded, and this was true for both species (t_412 _= 5.8, P < 0.001) and genera richness (t_412 _= 8.2, P < 0.0001) using Google Scholar citations, however, when using Zoological Record citations, the association was not significant. Zoological Record citations were affected by a number of outliers so this measure of sampling effort was excluded from further analyses.

**Table 1:** Host traits.

Variables	Charadriiformes	Pelecaniiformes	Procellariiformes
	(n = 241)	(n = 50)	(n = 122)
Body mass (g)	144	35	53
Wingspan (cm)	108	22	30
Body Size (cm)	145	31	64
Bill Length (mm)	73	12	54
Longevity (months)	112	21	29
Clutch Size(count)	110	35	46
Population Size (estimated numbers)	188	37	78
Geographic range (km^2^)	193	41	80
Diving behaviour	182	45	77

Variables used in the analyses with their respective sample sizes.

**Figure 1:**
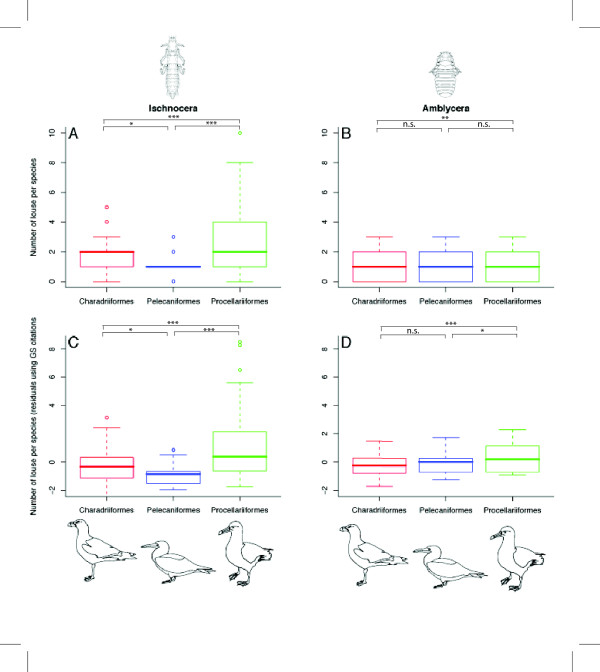
**Species diversity for each lice suborders**. Results are shown before controlling for sampling effort for (A) Ischnocera and (B) Amblycera and after controlling for sampling effort using Google Scholar (GS) citations for (C) Ischnocera and (D) Amblycera. Box plots show tenth, twenty-fifth, the median, seventy-fifth, and ninetieth percentiles, with points for outliers of these percentiles. The significant difference between the means are shown above the box plots (* < 0.05, ** < 0.01, *** < 0.001, n.s. = non-significant). Images of *Paraclisis diomedeae *(Ischnocera) and *Austromenopon affine *(Amblycera) obtained from [72] with permission from V. Smith.

We found that the average number of lice (Ischnocera and Amblycera combined) in the three different bird orders was significantly different (F_410,2_= 18.49, P < 0.01) before and after controlling for sampling effort using Google Scholar. The Procellariformes had significantly higher average taxonomic richness than the Pelecaniformes (t_164 _= -6.83, P < 0.0001) and the Charadriiformes (t_148 _= -6.04, P < 0.0001) when controlling for sampling. When comparing the Ischnocera diversity (0–10 Ischnocera per host) on the three bird orders, we found a significant difference (F_410,2 _= 6.17, P < 0.001, Fig 1A) with significantly more Ischnocera on Charadriiformes than Pelecaniformes and more Ischnocera on Procellariiformes than Charadriiformes. After controlling for sampling effort, there was still a significant difference in the average number of lice between the three bird orders (F_410,2 _= 23.95, P < 0.001, Fig 1C). In the Amblycera suborder (0–3 amblyceran lice per host) there was also a significant difference in the parasite species richness between the orders (F_410,2 _= 4.59, P = 0.01 Fig 1B and F_410,2 _= 17.57, P < 0.0001 after controlling for sampling effort, Fig 1D).

### Seabird phylogeny and phylogenetic patterning of variables

**Figure 2:**
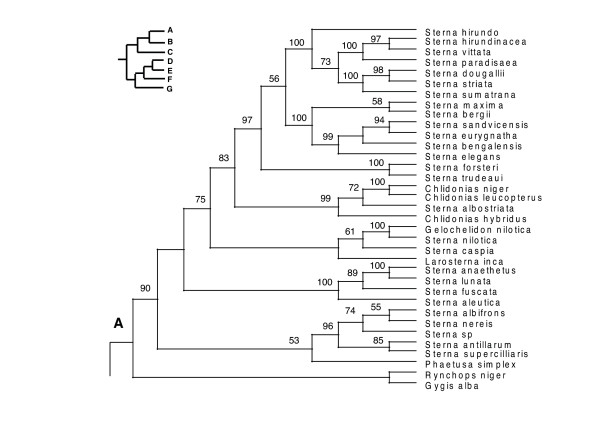
**Seabird phylogeny clade A**. One of 283 maximum parsimony phylogenies (Length 30618, CI = 0.185, RI = 0.602, RC = 0.111) with bootstrap values shown above the branches. This figure shows the upper quartile of the figure, for the full image please see Additional file 4.

**Figure 3:**
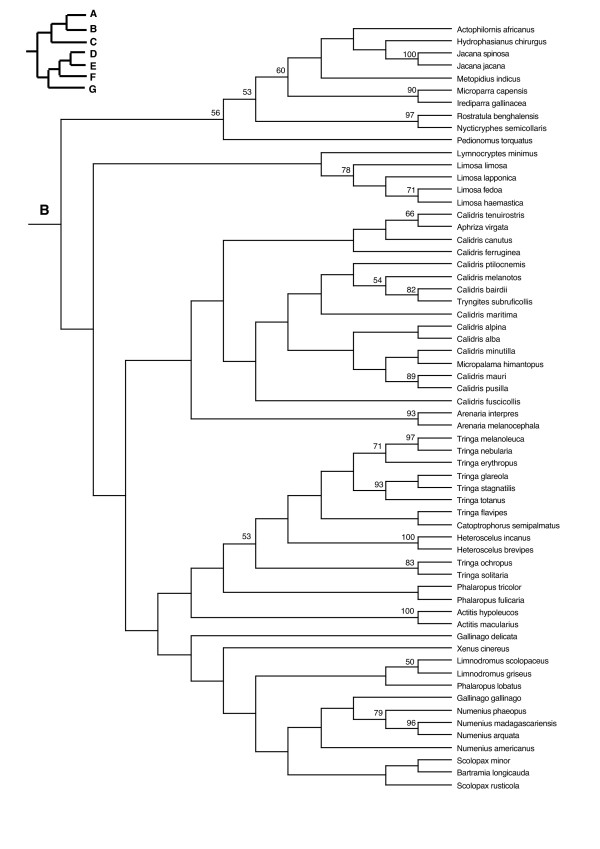
Seabird phylogeny clade B.

**Figure 4:**
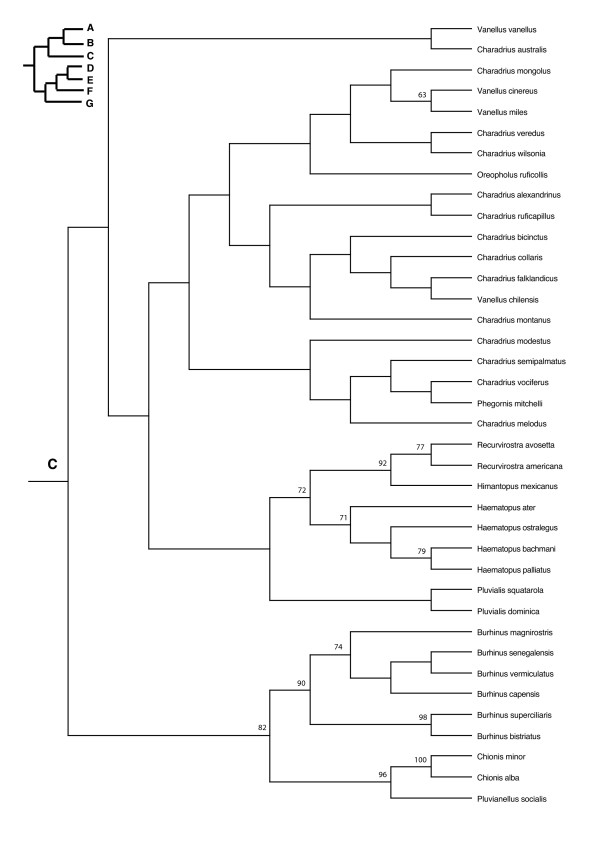
Seabird phylogeny clade C.

**Figure 5:**
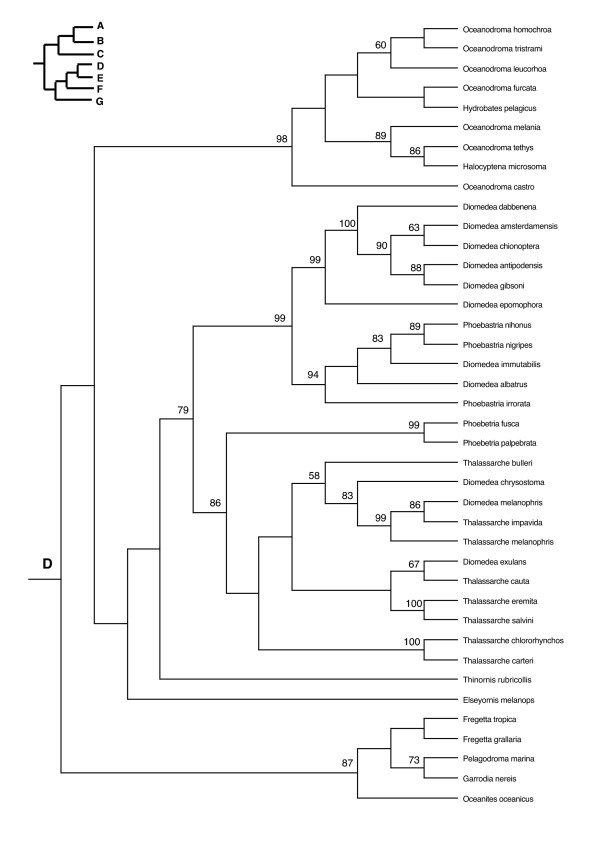
Seabird phylogeny clade D.

**Figure 6:**
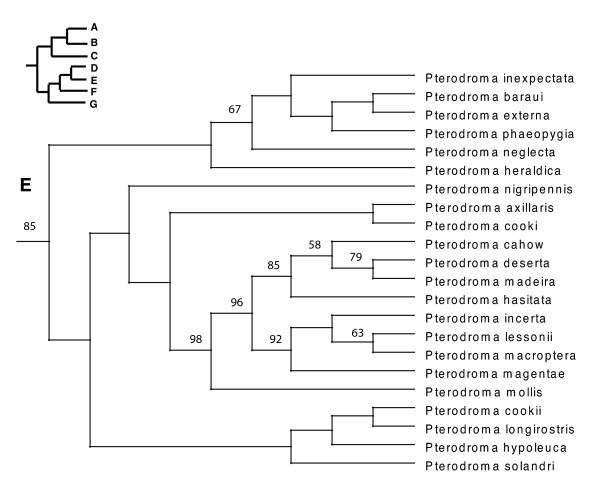
**Seabird phylogeny clade E**. This figure shows the lower quartile of the figure, for the full image please see Additional file 5.

**Figure 7:**
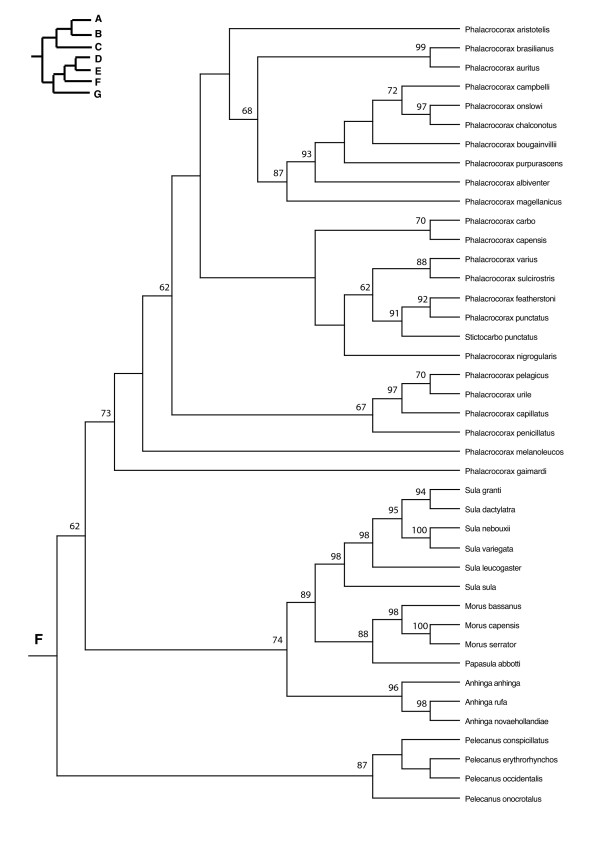
Seabird phylogeny clade F.

**Figure 8:**
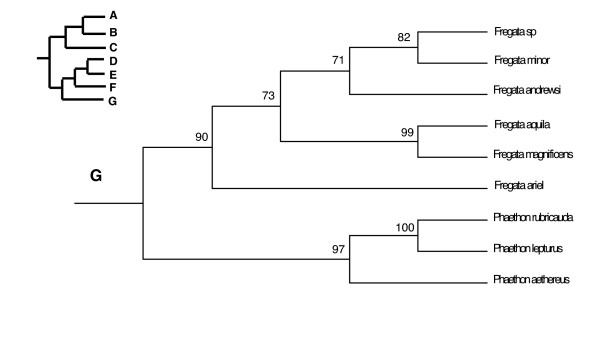
Seabird phylogeny clade G.

In the PAUP ratchet analysis, 283 out of 2000 trees had the shortest length of 30618 (CI = 0.185, RI = 0.602, RC = 0.111). The majority rule consensus of these trees was congruent with the family and subfamily level relationships of molecular studies of shorebirds (Charadriiformes) [38-40] except for the Stercorarini which is sister to the Alcinae in Thomas et al. [40], whereas here they form paraphyletic groups. The Pelecaniformes do not form a monophyletic order due to the paraphyly of the Phaethontidae as previously illustrated in Ericson [39] and discussed in greater detail in Kennedy and Spencer [41] and Kennedy et al. [42]. However, unlike the phylogeny of Kennedy and Spencer [41], the Fregatidae are sister to the Phaethontidae, perhaps as a result of long branch attraction [42]. The relationships within the Procellariiformes are also mainly congruent with previous studies [43]. Although the basal relationships are not highly supported by the bootstrap support, we prefer to use a fully resolved phylogeny for all comparative analyses, which we selected to be the most congruent with previous phylogenetic studies of seabirds (Figure 2, 3, 4, 5, 6, 7, 8, see also Additional Files 4 and 5). We take one of these phylogenies as a hypothesis open for future testing with additional data and new methods.

Tests of the correlation between parasite diversity and host phylogeny (λ tests) provided support for measures of PTR being associated among closely related host taxa. Prior to performing this test we controlled for sampling effort by regressing PTR measures on Google Scholar citation hits using a quadratic model (asymptotic); residuals from this analysis were then used as adjusted measures of PTR (r-PTR). All measures of r-PTR and of host traits showed some degree of phylogenetic patterning (λ > 0, Table 2). Given the significance of the phylogeny, both the non-phylogenetic and independent contrasts are reported. The focused (single predictor variable) tests are used to determine the sign of the relationship between the host traits and r-PTR; and to make certain our conclusions are robust, we focus on the findings where the phylogenetic and non-phylogenetic analyses yield the same result. As a number of the host traits might be non-independent or apparent only after controlling for other variables, we place more weight on the results from multivariate tests than from the focused tests and in particular multivariate tests calculated from contrasts.

**Table 2:** Lambda statistics for phylogenetic signal.

	Lambda	LRT	P-value
Residual Species	0.16	63.43	<0.001
Residual Genera	0.19	55.46	<0.001
Residual Ischnocera	0.18	63.53	<0.001
Residual Amblycera	0.28	58.08	<0.001
Body Mass	1.01	1978.46	<0.001
Wingspan	1.01	1300.92	<0.001
Body Size	1.04	2242.11	<0.001
Bill Length	1.07	1170.76	<0.001
Longevity	0.36	25.06	<0.001
Clutch Size	0.91	345.59	<0.001
Population Size	0.29	12.75	<0.001
Geographic Range	0.48	157.33	<0.001

All measures of parasite taxonomic richness and traits show significant phylogenetic signal. Measurements of parasite taxonomic richness are residuals from the regression of Google Scholar citation count against the measure of parasite richness. Significance of the likelihood ratio tests (LRT) was determined using the Chi-Squared distribution with 1 degree of freedom.

### Morphology

The colinearity test showed that all bird morphological variables were highly correlated (Table 3) but none of the life-history or ecological variables correlated highly with the morphological variables. Therefore, we carried out a principal component analysis with the morphological variables. The loadings shown in Table 4 suggest that principal axis 1 (PC1) is inversely related to overall size because all loadings are similar and negative. Hence, PC1 separates the larger birds from the smaller species. PC2 relates mainly to bill length, and PC3 separates the heavy birds with small wings to the light birds with large wings. PC4 principally relates to the body size. The positions of the different bird species on the four principal component axes (eigenvalues) were used for calculating the contrasts and in all multiple regression analyses, these axes all being independent from each other.

**Table 3:** Correlations between host traits.

	Body Mass	Wingspan	Body Size	Bill Length	Longevity	Clutch Size	Population Size	Geographic Range
	
Body Mass	1							
Wingspan	**0.88**	1						
Body Size	**0.95**	**0.91**	1					
Bill Length	**0.82**	**0.75**	**0.89**	1				
Longevity	0.45	0.52	0.51	0.52	1			
Clutch Size	-0.28	-0.36	-0.37	-0.23	-0.39	1		
Population Size	-0.24	-0.18	-0.19	-0.26	0.02	0.06	1	
Geographic Range	-0.34	-0.23	-0.27	-0.31	-0.04	0.61	0.52	1

Highly correlated variables are shown in bold (coefficient of correlation r = 0.6).

**Table 4:** Loadings from the principal component analysis.

	Comp.1	Comp.2	Comp.3	Comp.4
	
Body Mass	-0.509	-0.244	0.773	0.29
Wingspan	-0.501	-0.462	-0.621	0.387
Body Size	-0.52			-0.849
Bill Length	-0.468	0.849	-0.121	0.214

Loadings of the four log-transformed bird morphological variables (n = 73).

In focused tests (single predictor variables against r-PTR), residual species richness was not significantly correlated to body mass in non-phylogenetic analyses and negatively correlated in phylogenetic tests and so was body size in contrast to the positive relationship predicted (Table 5). PC3 (body mass/wingspan) did enter the multiple regression model with the highest log likelihood in non-phylogenetic (Table 6) and in phylogenetic analyses (Table 7). None of the morphological variables are significantly correlated to the residual genus richness in focused tests (Table 5) although the PC1-4 are present in the top three non-phylogenetic multiple regression models (Table 6) and PC1 enters the top three phylogenetic multiple regression models (Table 7). The residual Ischnocera richness is negatively correlated to body mass and body size in focused tests (Table 5) and PC1, PC3 and PC4 enter the top non-phylogenetic multiple regression model (Table 6). On the other hand, residual Amblycera richness is significantly correlated to wingspan in non-phylogenetic focused tests (Table 5) and negatively correlated to body mass in phylogenetic tests and all 4 PCs enter the non-phylogenetic (Table 6) and phylogenetic multiple regression models (Table 7). This suggests that morphology might play a more important role in predicting Amblycera species richness than Ischnocera richness. In general, it would appear that morphology does not play a consistent role in predicting the r-PTR and in the cases where body mass and/or body size do correlate to the r-PTR, the relationship is negative in contrast to our predictions.

**Table 5:** Regressions for non-phylogenetic and phylogenetic tests.

	Residual total species richness		Residual genus richness		Residual Ischnocera species richness		Residual Amblycera species richness	
				
	Actual Values	Contrasts	Actual Values	Contrasts	Actual Values	Contrasts	Actual Values	Contrasts
Body Mass		-				-		-
Wingspan							+	
Body Size		-				-		
Bill Length								
Longevity	+						+	
Clutch Size	-		-		-			
**Population Size**	**+**	**+**	**+**	**+**	**+**	**+**	**+**	**+**
Geographic range		+	-	+		+		+
Diving behaviour				+				

Simple regressions for single variables controlled for sampling effort using the residuals from Google Scholar citation counts. Signs indicate whether the slope was negative or positive.

**Table 6:** Multiple regression results.

	Non-phylogenetic analyses			
	log Lik	AIC	Δ_i_	w_i_
	
Residual Species Richness				
Long*, ClutchSize*, GlobPop*, GeoRange, PC3, PC4	-116.02	252.04	0.00	0.42
Long*, ClutchSize*, GlobPop*, GeoRange, PC1	-117.49	252.98	0.94	0.26
Long*, ClutchSize*, GlobPop*, GeoRange, PC1, PC3, PC4	-115.80	253.61	1.57	0.19
Residual Genus Richness				
Long, ClutchSize*, GlobPop*, GeoRange*, PC3, PC4	-84.63	199.27	0.00	0.42
Long, ClutchSize*, GlobPop*, GeoRange*, PC1, PC3, PC4	-89.60	201.21	1.94	0.16
Long, ClutchSize*, GlobPop*, GeoRange*, PC1, PC2, PC3, PC4	-89.32	202.64	3.37	0.08
Residual Ischnocera Richness				
Long, ClutchSize*, GlobPop*, GeoRange, PC3, PC4	-99.70	219.41	0.00	0.42
Long, ClutchSize*, GlobPop*, GeoRange, PC1	-100.71	219.43	0.02	0.42
Long, ClutchSize*, GlobPop*, GeoRange, PC1, PC3, PC4	-99.30	220.61	1.2	0.23
Residual Amblycera Richness				
Long*, ClutchSize, GlobPop*, GeoRange, PC3*, PC4	-55.68	131.35	0.00	0.42
Long*, ClutchSize, GlobPop*, GeoRange, PC1, PC3*, PC4	-55.68	133.35	2.00	0.16
Long*, ClutchSize, GlobPop*, GeoRange, PC1, PC2, PC3*, PC4	-54.83	133.67	2.32	0.13

Log Likelihood values (log Lik), Akaike's information criteria (AIC), change in AIC score (Δ_i_), and AIC weight (w_i_) for the top four of 26 candidate models relating host longevity (Long), estimates of global population size (GlobPop), geographic range (GeoRange), clutch size (ClutchSize) and the four principal component (see Table 4) to the raw values of parasite taxonomic richness (PTR). Sampling effort (Google Scholar citations) was included as an asymptotic variable in all the models shown in this table by using the residuals. * denote variables that were significant in the focused tests.

### Life history

Residual species richness was significantly correlated to longevity and clutch size in non-phylogenetic focused tests (Table 5) and in non-phylogenetic multiple regressions (Table 6) but was no longer significantly correlated when the phylogeny was taken into account (Table 5 and Table 7). Residual genus richness was significantly correlated to clutch size in non-phylogenetic focused tests (Table 5) and in both multiple regression tests while longevity was only a significant predictor in multiple regression tests (Table 6 and 7). Clutch size was also significantly negatively correlated with residual Ischnocera richness, although clutch size did not enter the top phylogenetic multiple regression analyses (Table 7). Whereas longevity was correlated positively to residual Amblycera richness in non-phylogenetic focused tests (Table 5). Longevity and clutch size were found in both multiple regression analyses for residual Amblycera richness (Table 6 and 7). Thus, longevity and clutch size appear to be predictors for residual genus richness and residual Amblycera richness but not species richness and Ischnocera richness. Longevity was positively correlated with genus richness and Amblycera richness as expected whereas clutch size was negatively correlated contrary to our predictions.

**Table 7:** Multiple regression results controlling for phylogeny.

	Phylogenetic analyses			
	log Like	AIC	Δ_i_	w_i_
	
Contrast of Residual Species Richness				
PC3	-126.01	260.03	0.00	0.30
PC1*, PC3	-125.94	261.88	1.85	0.12
GlobPop*	-127.62	263.24	3.21	0.06
Contrast of Residual Genus Richness				
Long	-106.85	221.71	0.00	0.21
Long, ClutchSize	-106.26	222.53	0.82	0.14
Long, PC1	-106.72	3.45	1.74	0.09
Contrast of Residual Ischnocera Richness				
GlobPop*, GeoRange*	-111.33	232.67	0.00	0.13
GeoRange*	-112.38	232.77	0.10	0.12
GlobPop*	-112.72	233.44	0.77	0.09
Contrast of Residual Amblycera Richness				
Long, ClutchSize, GlobPop*, GeoRange*, PC3, PC4	-72.03	162.06	0.00	0.28
Long, ClutchSize, GlobPop*, GeoRange*, PC1*, PC2, PC3, PC4	-70.41	162.82	0.76	0.19
Long, ClutchSize, GlobPop*, GeoRange*, PC1*, PC3, PC4	-71.55	163.11	1.05	0.16

Log Likelihood values (log Lik), Akaike's information criteria (AIC), change in AIC score (Δ_i_), and AIC weight (w_i_) for the top four of 25 candidate models relating host longevity (Long), estimates of global population size (GlobPop), geographic range (GeoRange), clutch size (ClutchSize) and the four principal component (PC1-4, Table 4) to the independent contrasts of parasite taxonomic richness (PTR). Sampling effort (Google Scholar citations) was included as an asymptotic variable in all the models shown in this table. * denote variables that were significant in the focused tests.

### Total population size and geographic range

**Figure 9:**
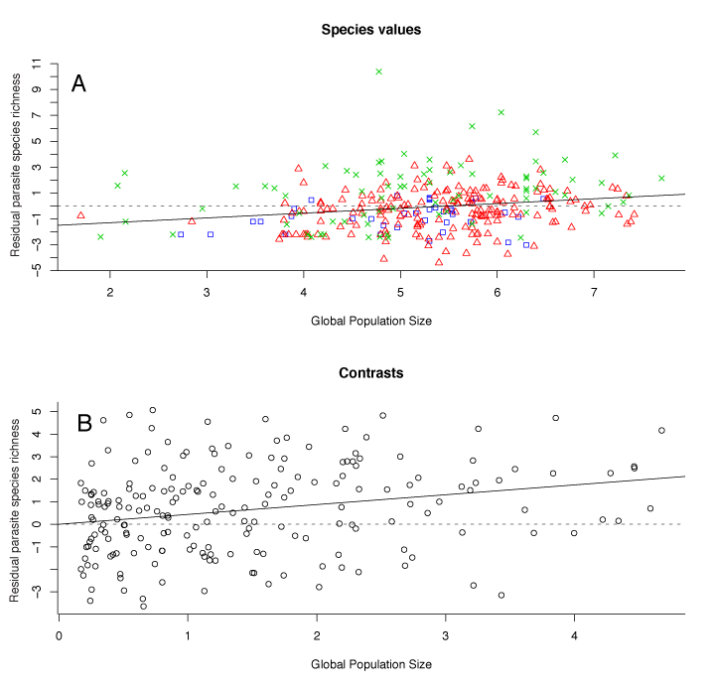
**Regression between global population size and overall lice species richness**. Lice species richness was controlled for sampling effort using residuals from the Google Scholar citation counts. A, Regression for non-phylogenetic analysis of species values (slope = 0.41, F = 16.82, d.f. = 301, P < 0.0001). Pelecaniiformes are represented by blue squares, Charadriiformes by red triangles and Procellariiformes by green crosses. B, Regression using independent contrasts (slope = 0.43, F = 33.4, d.f.= 196, P < 0.0001).

Population size offers the most compelling results as it is positively correlated to all measures of r-PTR in focused tests (Table 5) as expected and appears in the top models for all multiple regression analyses except for genus richness when controlling for phylogeny (Table 6 and 7 and Figure 9). Geographic range is positively correlated in phylogenetic focused tests (Table 5) but negatively correlated to residual genus richness in the phylogenetic focused test. Geographic range also appears to be an important predictor as it is significantly positively correlated to all measures of r-PTR in phylogenetic focused tests (Table 5) and in all non-phylogenetic multiple regression models (Table 6). Geographic range is also a predictor of Ischnocera richness and Amblycera richness in phylogenetic multiple regressions (Table 7).

### Diving behaviour

Diving behaviour was a significant predictor of residual genus richness in phylogenetic focused tests. Significantly more genera were found on non-diving birds as expected but this correlation was not significant for any of the other measures of r-PTR.

### Correlation between host diversification and parasite taxonomic richness

The different measures of r-PTR were not significantly correlated with the evolutionary diversification of seabirds as determined in MacroCAIC. Thus, parasite diversity (louse taxa per host species) did not differ between birds from relatively diverse clades compared to the less diverse clades. The results were the same for all measures of r-PTR and for both measures of host diversification (RRD and PDI, Table 8). These results are also supported by the lack of significant relationships between bird order and r-PTR in the multiple regression.

**Table 8:** Association between host diversification and parasite richness

	RRD		PDI	
	slope	F-ratio	slope	F-ratio
	
Residual Species	-0.0043	0.1240, n.s.	-0.0008	0.1218, n.s.
Residual Genera	-0.0084	0.1735, n.s	-0.0014	0.1336, n.s.
Residual Ischnocera	-0.005	0.0904, n.s.	-0.0009	0.0891, n.s.
Residual Amblycera	-0.0142	0.1481, n.s.	-0.0027	0.1444, n.s.

All analyses included 275 contrasts. RRD = relative rate difference, PDI = proportional dominance index, n.s. = not significant.

To summarise the results, it would appear that morphological characteristics such as mass, size, wingspan and bill length play a minor role in predicting the r-PTR and the patterns observed are inconsistent across the different measures of r-PTR. Morphological traits may play a greater role in predicting Amblycera richness than Ischnocera richness. Longevity and clutch size are predictors in residual Amblycera and genus richness but the results are not as compelling for Ischnocera and total species richness. Population size and to a lesser extent geographic range are the predictors that show the most consistent correlations to all measures of r-PTR providing the most compelling support for the role of these two variables as predictors of parasite richness. Diving behaviour was only correlated in the phylogenetic test for residual genera richness. And finally, the diversification of birds was not correlated to any of the measures of parasite richness.

## Discussion

As discussed elsewhere [21,32] differential sampling effort must be taken into account when investigating parasite community structure. In the present study, correction for variation in the number of citations of parasitological studies using Google Scholar citations for each host species provides perhaps the most pragmatic method of control of potentially confounding effect. The inclusion of the phylogeny was also critical for testing the evolutionary hypotheses as demonstrated by the correlation between the phylogeny and the various host traits and the r-PTR measures.

Bird morphology and mass were not consistently correlated to parasite species richness in our study although body mass and size did enter into the multivariate regression models for some measures of r-PTR. This contrasts with a number of previous studies that have recognized host body size as an important determinant of parasite richness [4,9,44,45]. Larger-bodied hosts may represent larger islands for parasites to colonize, suggesting that more parasites will be found on larger hosts. Indeed, a number of mammalian studies found strong positive relationships between body mass and parasite species richness [1-3] but other studies on birds also found that host body size showed no relationship with parasite species richness [8] leading to the suggestion that the correlation between host body size and parasite community richness may vary between certain host groups [8]. Additionally, the same patterns are not always observed across different taxonomic groups of parasites, for example, Gregory et al. [45] found a positive correlation between host weight and the number of trematode and nematode species, but not cestodes. We also found slight differences between the two parasite groups studied (Amblycera and Ischnocera) with morphology playing a greater role in Amblycera species richness as in a previous study [10]. Thus, the role of body mass and size as correlates of lice species diversity in seabirds cannot be entirely dismissed but these variables do not appear to be as important as in mammals for predicting parasite species richness.

The life-history traits (longevity and clutch size) were present in the top phylogenetic multiple regressions for Amblycera richness suggesting that species richness of Amblycera is more closely correlated to host traits than in the Ischnocera. The lack of significant morphological and life history correlates for Ischnocera species richness in the multiple regression as opposed to the Amblycera seems to be inherent to the suborder. Neither Møller and Rozsa [10] nor Clayton and Walther [9] found significant correlates for the Ischnocera species richness. Correlates of species diversity in the Ischnocera may be more difficult to discover than those of amblyceran lice perhaps due to the differences in feeding behaviour of the two suborders, although this hypothesis is highly speculative. Amblycera come into direct contact with their host when feeding on skin or blood leading to a correlation between the immune response of birds and Amblycera richness [10] and perhaps also the correlation we found here with longevity and clutch size. On the other hand, Ischnocera lice feed on the keratin of feathers, thus indirectly affecting their host through feather damage. As the feather represents the niche of an Ischnocera louse, perhaps the diversity of Ischnocera lice will change with the diversity of feather types on a host, although this host trait is unlikely to vary sufficiently within bird families to detect significant relationships at a species level.

By placing the most confidence on multivariate tests that take into account the non-independence of variables, the strongest results emerged from analyses of the effects of population size with all four measures of taxonomic richness (ischnoceran, amblyceran, overall species richness and genus richness). Our analyses thus suggest that epidemiological processes operating within a species provide explanations for broad patterns of parasite biodiversity. Indeed, hosts with large population sizes may influence the acquisition of parasite species as they may be more likely to come into contact with conspecifics and thus facilitate the spread of the parasite through the population and influence the size of the parasite community through island biogeographic effects (a larger population representing a larger island for colonization by the parasite). Moreover, species with greater geographical ranges also have greater parasite richness and this might be because they come into contact with a greater number of habitats and parasite species. Thus, as predicted, species with larger population sizes and geographical ranges have more parasites. However, the positive correlation between geographic range and parasite richness could be a result of sampling bias that was not controlled for. Indeed, it is possible that parasite richness is underestimated in non-social territorial birds compared to colonial birds as lice are more aggregated in non-social birds [46] and therefore the sampling of lice on territorial birds which usually have larger geographical ranges is likely to be less complete. Thus, the positive relationship between host geographic range and parasite richness could either be a true effect of island biogeography, a sampling bias inadequately controlled for or, most probably, the sum of both.

Diving behaviour also explains parasite richness but only in the case of genera richness in the phylogenetic test, these results support a previous study on correlates of parasite richness with diving behaviour [34] but it is interesting to note that the same pattern was not observed for the other three measures of parasite richness (total species richness, Amblycera richness and Ischnocera richness). Using phylodiversity as a measure of parasite richness would help to determine whether these differences are caused by the over-estimation of species due to uncertain morphological taxonomy.

Although most studies on lice have focused on patterns of cospeciation within small clades of hosts and parasites and have found strong evidence for cophylogeny [11,13,35], in this study looking at the broader pattern of host diversity in relation to parasite diversity, we did not find a correlation between host diversification and parasite species richness. Thus, it appears that diversification of seabird lineages do not provide greater opportunities for increasing parasite species richness, unlike studies of host diversification in primates that found a strong positive relationship between parasite richness and host diversification [3]. Further studies on a broader range of birds and parasites (including microparasites) would be helpful to gain a better understanding of the relationship between bird diversification and parasite richness.

Unfortunately, the variables used in this study do not always explain the variation in parasite richness in phylogenetic tests. In particular, the Akaike weights of the multiple regression models in phylogenetic tests is not very high and can be interpreted as a low probability that the models for the phylogenetically corrected data is the 'true' model. This could indicate that we have not included the variables crucial to understanding the diversity of ectoparasites, i.e. that different ecological processes not included here affect the ability of different ectoparasites to establish. This could also be caused by the noise in the data as a result of the indirect measurement of sampling effort (i.e. citation counts) being used. Nonetheless, the approach taken in this study provides the best inference given the data and the set of a priori models. Akaike's general approach allows the best model in the set to be identified, but also allows the rest of the models to be easily ranked. New or more elaborate hypotheses can be added in the future and hypotheses with little empirical support can gradually be dropped from consideration.

A number of issues need to be raised with regards to the data. Firstly, the species richness measures do not represent parasite communities, as they are the sum of all parasite species found in several host populations; thus, there may be no single population of host where all parasite species would co-occur. Studying the parasite species recorded for different host species in one geographical location was not possible here due to insufficient information in the literature relating to collection location. Secondly, the data on parasite richness are unlikely to be of uniform quality for the different hosts, due mainly to the diversity of methods used by different investigators. This effect could not be controlled for in this study, but it probably would not have much of an impact on the results. The latter two issues have successfully been taken into account in a study of Neotropical bird lice [9]. Clayton and Walther [9] sampled using consistent methodologies in a single geographic region and did not find any correlation between louse species richness and any of the 13 host variables examined (including host body size, density and geographic range). This could be a result of controlling for phylogeny and sampling effort or the small variation in louse species richness (from 0–3 species). Variation in species richness in our study is unlikely to be a problem for the overall lice species richness (0–13 species), genus richness (0–7) and Ischnocera richness (0–10) but might have been for the Amblycera richness (0–3) as it is more difficult to detect correlations involving variables that show little variation.

## Conclusion

Broad patterns of parasite diversity were explained by a relatively small number of host characteristics, especially host population size and geographic range and differences were observed between the two louse suborders. In particular, morphology and life-history are a better predictor of Amblycera richness than Ischnocera richness. Further details on bird ecology will allow us to investigate the role of a larger number of predictor variables. For example, further research is needed to gather data such as local population density, home range and daily journey length, which could help to account for further variation among host species. We also need to determine whether parasite species richness is higher as a result of greater opportunities for host sharing or host shifting among sympatric seabirds. For this we need precise geographical range maps and measures of overlap. Increased overlap could increase opportunities for specialist parasites to host shift. Since many ectoprarasitic lice of birds are highly host specific [47], the richness of parasite communities may depend more on which parasite lineages host shift, than on evolutionary changes in host size or habitat. One approach for resolving this would be to examine variation in the host specificity for particular lice species using host phylodiversity. A better predictor of parasite species richness remains to be discovered and it may be that this understanding can be achieved only by gathering detailed data of lice communities on individual hosts (e.g., wing versus body versus head lice) rather than the much broader analysis using species richness.

## Methods

### Taxonomic richness of lice and controlling for sampling effort

Four different measures of parasite taxonomic richness (PTR) were compiled from the world checklist of chewing lice [47] available from BioCorder [48] for 413 bird species (Charadriiformes, Pelecaniformes, Procellariiformes): Ischnoceran species richness, Amblyceran species richness, overall parasite species richness and genera richness. To control for uneven sampling effort in estimating PTR, we followed previous researchers [2,10,32,49]. The record of a parasite on a host species may be missing in the literature either because it does not occur on that host or because the parasitic fauna of the host has been insufficiently sampled [32,49]. To determine how well a species had been studied for parasites, sampling effort was estimated in two ways. First, we assessed the intensity of parasitological surveys focused on different bird species using the citation index in the Web of Science Zoological Records (ZR). The number of hits on host scientific name mentioned with any of the terms "parasit*", "pathogen*", "helminth*", "mite*", "louse", "lice" was used as a measure of sampling effort (where "*" acts as a truncation sign). Second, we used the number of hits on host scientific name and "mite OR mites OR parasite OR parasites OR parasitic OR helminths OR helminth OR lice OR louse" in Google Scholar (GS), that does not allow truncation signs. Google Scholar provides all unique citations from books, journals and reports (different versions of the same reference were not included in the citation count). The asymptotic model between the citation index and the PTR (parasite taxonomic richness) is expected to be better than a linear model. This was tested using the Akaike Information Criterion as a measure of the model fit and only significantly correlated measures of citation were used in further analyses. Thus, we included sampling effort by calculating the residual PTR, i.e. regressing the parasite richness on measures of sampling effort, according to the best fit model. The residual PTR was then used in all further analyses.

### Data on bird characteristics

Using existing compilations of data [50-52] as a starting point, we created a database of comparative data on birds, which is available (together with details on sources of data) at [53]. We included the following morphological variables: body mass, wingspan, body size (bill-tip to tail-tip) and bill length. Longevity is one of the key factors that could influence parasite diversity and was measured as maximum recorded longevity in months with most of the data retrieved from Carey and Judge [52] and clutch size was the average size from the different data sources. Unfortunately, measures of social contact within and between species such as local population density are not easily obtainable for seabirds. Thus, using Bird Life International [54], we gathered estimated global population size (number of individuals) and estimated geographic range in square kilometres. Diving behaviour information was also gathered as a categorical variable from a number of sources including a previous comparative study [34]. All continuous host characteristics were log-transformed and checked for collinearity to avoid adding collinear variables into the same model using a conservative cut-off value (correlation coefficient r = 0.6). We conducted a principal component analysis on variables that were strongly correlated and used the eigenvalues of each species on the independent principal component axes for all multiple regressions. The distribution of the log-transformed population size and geographic range was compared to the normal distribution with the Kolmogorov-Smirnov test to assess biases in the data.

### Comparative methods

Although parasite taxonomic diversity and the measures of sampling effort might not be shared through common descent in birds, they may be correlated with other host characteristics that are inherited (e.g. body size). Thus, traits for species cannot be considered statistically independent observations because cases of convergent evolution are mixed with cases of similarity due to common ancestry and thus phylogeny cannot be ignored [55-57].

Thus, we used a phylogeny built from all the available nucleotide sequences on NCBI extracted on the 30^th ^of November 2006. A supermatrix of the data was constructed using TaxMan [58] where the following genes were concatenated for 407 taxa: 1041 bases of CytB, 695 bases of 12S rRNA, 1551 bases of COX1, 684 bases of ATPase6, 1047 bases of NADH2 and 2871 bases of RAG1. We implemented parsimony ratchet searches [59,60] using PAUP [61] by performing 10 independent Parsimony Ratchet searches of 200 iterations each. Bootstrapping 100 heuristic replicates was used to determine the level of support for individual nodes.

We assessed whether phylogenetic correction was needed for our data by calculating Pagel's λ statistic for each measure of PTR and for each host trait using the software program Continuous implemented in BayesTraits [62-64]. The λ statistic tests whether a trait is evolving among species as if the species were independent (λ = 0) by determining if phylogeny correctly predicts patterns of covariance among species. We used a likelihood ratio test to compare the maximum likelihood estimate of lambda for each trait to a lambda estimate of zero, and assumed phylogenetic patterning when the lambda estimate for a trait was significantly different to zero.

We calculated statistically independent linear contrasts for each variable according to the method developed by Felsenstein [65] using the computer program CAIC [66]. We also performed non-phylogenetic analyses using actual species values as comparisons of phylogenetic and non-phylogenetic results can reveal the presence of confounding factors [67]. The analyses were carried out using log-transformed data and the branch lengths estimated in PAUP, i.e. assuming a gradual evolution model as implemented by Purvis and Rambaut [66] against the four different measures of parasite richness controlled for sampling effort (r-PTR). Continuous variables were analysed using crunch and the categorical variable "diving behaviour" was analysed with brunch.

First, we conducted analyses of all single predictor variables against the PTR controlled for sampling effort using residuals from the regression of the number of parasites for each host on the number of citation counts (focused tests). Contrasts were calculated for the residual PTR and the log-transformed variables.

Second, we used multiple regression to investigate the continuous variables that explain variation of r-PTR: the morphological principal components and life-history and ecological traits (population size, longevity, clutch size and geographic range). In phylogenetic tests, the contrasts were calculated for the r-PTR and principal components prior to multiple regression analyses. The generalised linear model for the contrasts was forced through the origin. We fitted models to our data set using 'glm' in R and compared them using AIC rather than using a stepwise technique which has widely recognized limitations [68]. AIC is a likelihood-based measure of model fit that accounts for the number of parameters estimated in a model, (i.e. models with a large number of parameters are penalized more heavily than the ones with smaller numbers of parameters). The model with the lowest AIC has the 'best' relative fit, given the number of parameters included [69]. We tested a model with all the variables, a model with just the morphological variables, one with just the life-history traits (longevity and clutch size), one with just the population and distribution variables (population size and geographic range), a model with life-history traits, population and distribution variables, a model with all the significant variables form the focused tests and models with each single predictor variable (8 models) and in non-phylogenetic tests, bird order was also tested as a predictor of parasite species richness. In order to control for correlations between body mass and other traits, the first principal component (with high body mass loading) was forced into the models if it had not already been included (11 models). Thus, 26 models for non-phylogenetic data and 25 models for phylogenetic data were tested for each measure of PTR (see Additional File 3 for the list of models). The plausibility of these competing statistical models was then assessed using penalized log likelihood criteria (AIC = -2ln L + 2k, L being the maximum likelihood estimate and k the number of free parameters). The raw AIC values were then transformed to AIC model weights as in Burnham [70]. Akaike weight, w_i_, provides the weight of evidence in favor of a model i according to:

$$w_{i}=\frac{exp(-\Delta_{i}/2)}{\sum_{r=1}^{R}exp(-\Delta_{r}/2)}$$

where Δ_i _= AIC_i _- minAIC.

To test whether there is an association between parasite species richness and host lineage diversity, we used the computer program MacroCAIC 1.1.1 [71]. This program generated phylogenetically independent contrasts for PTR (controlling for sampling effort) at each node with three or more descendants and compares this to the number of host species within the clade represented at each node. We used the two measures of host phylogenetic diversity available: the relative rate difference (RRD) calculated as the natural logarithm of the ratio of the number of species in sister clades; and, the proportional dominance index (PDI) calculated as the ratio of the number of species in one of two clades against the total number of species in both clades combined [71].

## Authors' contributions

JH acquired the data, carried out the analyses and drafted the manuscript. RDMP wrote scripts for gathering and parsing data and participated in critical revision. JH was supported by NERC grant NE/B000079/1 to RDMP. Both authors read and approved the final manuscript.

## Supplementary Material

Additional file 1 **Nexus matrix and phylogeny**. Sequence alignment of seabirds (Charadriiformes, Pelecaniformes and Procellariiformes) used for building the phylogeny in the nexus format. The majority rule and ultrametric phylogenies can be visualised in Treeview. Click here for file

Additional file 2 **Morphological, ecological and parasite richness data**. The morphological measures include body mass (grams), body size (centimetres), bill length (millimetres) and wingspan (centimetres). The ecological variables are global population size (number of individuals), maximum longevity (months), geographic range (in km square), clutch size (average). The latter measures are log-transformed averages. Parasite richness measures are total number of species, Ichnocera species, Amblycera species and total number of genera. Diving behaviour is included as a categorical variable [1 = diving, 2 = non diving]. The raw data and associated references can be obtained from [53]. Click here for file

Additional file 3 **Multivariate models tested**. List of models tested in R with actual values (non-phylogenetic) and contrasts (phylogenetic). Click here for file

Additional file 4 Seabird phylogeny complete clade A. Click here for file

Additional file 5 Seabird phylogeny complete clade E. Click here for file
