# Supplementary material for: Comparative tests of ectoparasite species richness in seabirds
Source: BMC Evol Biol. 2007 Nov 15;7:227. doi: 10.1186/1471-2148-7-227 (PMC2258205; doi:10.1186/1471-2148-7-227)
Supplement: Additional file 3 — Multivariate models tested. List of models tested in R with actual values (non-phylogenetic) and contrasts (phylogenetic). [file 1471-2148-7-227-S3.doc]

List of non-phylogenetic models

| Longevity + ClutchSize + GlobalPop + GeoRange + PC3 + PC4 |
| --- |
| Longevity + ClutchSize + GlobalPop + GeoRange + PC1 + PC3 + PC4 |
| Longevity + ClutchSize + GlobalPop + GeoRange + PC1 + PC2 + PC3 + PC4 |
| Longevity + ClutchSize + GlobalPop + GeoRange + PC1 |
| Longevity + ClutchSize + PC1 |
| GlobalPop + GeoRange + PC1 |
| Longevity + PC1 |
| GlobalPop + PC1 |
| ClutchSize + PC1 |
| GeoRange + PC1 |
| PC3 |
| PC1 + PC3 |
| PC1 + PC2 + PC3 + PC4 |
| PC1 |
| PC4 |
| PC2 |
| PC1 + PC4 |
| PC1 + PC2 |
| Longevity + ClutchSize + GlobalPop + GeoRange |
| Longevity + ClutchSize |
| Longevity |
| ClutchSize |
| GlobalPop + GeoRange |
| GlobalPop |
| GeoRange |
| Order |

List of models fitted on the contrasts (forced through the origin)

| Longevity + ClutchSize + GlobalPop + GeoRange + Comp1 + Comp2 + Comp3 + Comp4 - 1 |
| --- |
| Longevity + ClutchSize - 1 |
| GlobalPop + GeoRange - 1 |
| Longevity + ClutchSize + GlobalPop + GeoRange - 1 |
| PC1 + PC2 + PC3 + PC4 - 1 |
| Longevity + ClutchSize + GlobalPop + GeoRange + PC3 + PC4 - 1 |
| Longevity - 1 |
| ClutchSize - 1 |
| GlobalPop - 1 |
| GeoRange - 1 |
| PC1 - 1 |
| PC2 - 1 |
| PC3 - 1 |
| PC4 - 1 |
| Longevity + ClutchSize + PC1 - 1 |
| GlobalPop + GeoRange + PC1 - 1 |
| Longevity + ClutchSize + GlobalPop + GeoRange + PC1 - 1 |
| Longevity + ClutchSize + GlobalPop + GeoRange + PC1 + PC3 + PC4 - 1 |
| Longevity + PC1 - 1 |
| ClutchSize + PC1 - 1 |
| GlobalPop + PC1 - 1 |
| GeoRange + PC1 - 1 |
| PC1 + PC2 - 1 |
| PC1 + PC3 - 1 |
| PC1 + PC4 - 1 |
